# Rapid detection of the beech leaf nematode, *Litylenchus crenatae* using recombinase polymerase amplification assay with lateral flow dipsticks

**DOI:** 10.1371/journal.pone.0341967

**Published:** 2026-02-09

**Authors:** Mihail Kantor, Sergei A. Subbotin

**Affiliations:** 1 Plant Pathology & Environmental Microbiology Department, The Pennsylvania State University, University Park, Pennsylvania, United States of America; 2 Plant Pest Diagnostic Center, California Department of Food and Agriculture, Sacramento, California, United States of America; University of Florida Tropical Research and Education Center, UNITED STATES OF AMERICA

## Abstract

Beech leaf nematode, *Litylenchus crenatae*, is the causal agent of beech leaf disease (BLD), a condition characterized by interveinal dark-green banding, leaf thickening, bud abortion, and potentially tree mortality. Current identification methods for *L. crenatae* rely on morphometric analysis or molecular techniques such as conventional PCR and real-time PCR. To support timely management of BLD, there is a need for a rapid, field-deployable detection method. Recombinase polymerase amplification (RPA) is a new isothermal *in vitro* nucleic acid amplification technique that has been adopted for rapid and reliable diagnostics of nematodes. In this study, RPA assay combined with lateral flow (LF) dipsticks has been developed targeting the ITS rRNA gene of *L. crenatae*. The assay demonstrated high specificity, sensitivity, enabling direct detection from crude nematode extracts and from DNA leaf plant extracts. Assay specificity was validated against a range of non-target nematode species. The LF-RPA assay showed reliable detection within 28–30 min with a sensitivity of 0.002 nematode per reaction tube for crude nematode extracts or 0.03 nematode per reaction tube using DNA extracts from leaves. The LF-RPA assay presents a practical diagnostic tool for plant clinics and forestry professionals, enabling rapid on-site detection of *L. crenatae* in infested beech trees to support timely disease management decisions.

## Introduction

**Fig 1:**
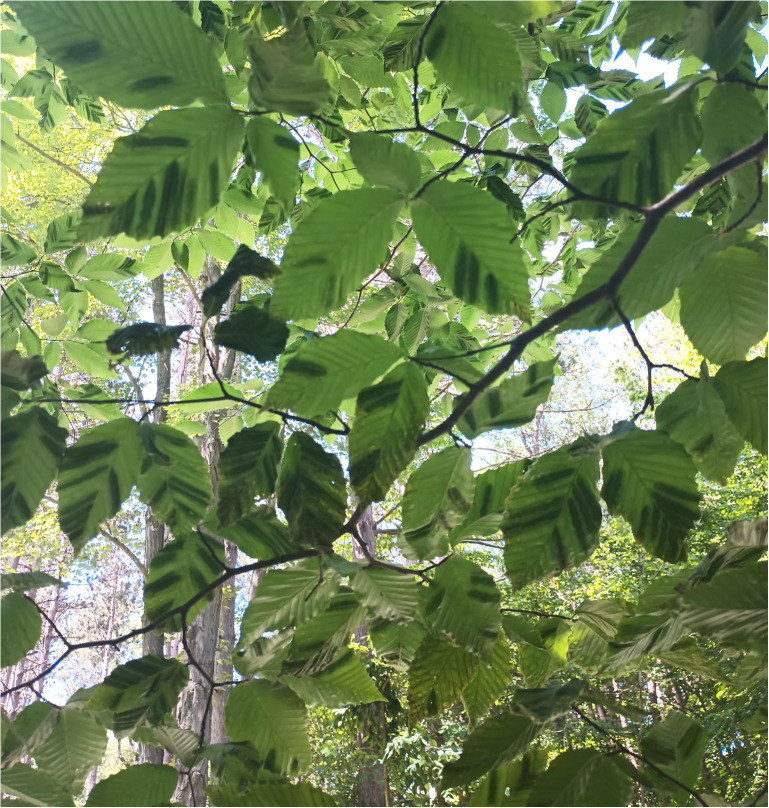
Symptoms of beech leaf disease in a forest, Pennsylvania, USA.

Beech leaf disease is the fastest spreading disease currently known, caused by a nematode, more specifically, *Litylenchus crenatae* [1,2]*.* Since its first report from Lake County, OH, in 2012 [3], this disease has rapidly spread to 14 other U.S. states and Ontario Province, Canada [4]. A recent study conducted by Goraya et al*.* [5] showed that environmental factors such as rain and wind play a crucial role in their rapid dispersal at the local scale. In addition to the environmental factors, these nematodes may be dispersed by a variety of beech forest inhabitants, such as insects, birds, and mammals, by hitchhiking on them as incidental vectors [4–6]. This disease has severe effects on American beech and is known to cause young tree mortality within 7 years of infection [7]. Owing to its destructive impact on American beech, this nematode has been listed as a quarantine candidate by the European and Mediterranean Plant Protection Organization since 2019 [4]. Despite the fact the BLD symptoms are easily recognizable (Fig 1) and include discoloured dark-green interveinal bands of the leaves [3], misidentification remains common.

Beech leaf nematode can be identified through morphological characteristics, which require highly trained experts, and by using different molecular techniques such as conventional and real-time PCR with species-specific primers. Burke et al. [8] developed a set of DNA primers for conventional and real-time PCR targeting the internal transcribed spacer (ITS) region of the rRNA gene that specifically amplified *L. crenatae* DNA. Vieira et al. [2] made the detection of *L.* *crenatae* with a real-time PCR and primers targeting a fatty acid- and retinol-binding gene (FAR). Molecular methods of identification require the use of laboratory space, specialized equipment, and sometimes lengthy DNA extraction protocols. Early and rapid detection of the beech leaf nematode is important for monitoring the spread of this pest.

Recombinase polymerase amplification (RPA) is a new isothermal in vitro nucleic acid amplification technique that has been successfully adopted for simple, robust, rapid and reliable diagnostics of nematode pests [9]. RPA offers a highly adaptable and effective alternative to PCR [10,11]. This technique employs a highly efficient strand-displacing polymerase to amplify a minimal amount of target nucleic acid within approximately 20 minutes at a constant temperature ranging from 37^o^C to 42^o^C [12]. RPA presents several advantages over conventional PCR-based methods for nematodes detection, most importantly, RPA operates under isothermal conditions, eliminating the need for thermal cycling and making it suitable for use in forest environments, with minimal infrastructure and limited technical expertise.

This study describes the development of and validation of RPA assay for rapid and specific detection of *L. crenatae* from BLD symptomatic leaves, nematode DNA, as well as crude nematode extracts. Species-specific primers and probe were designed based on the sequence polymorphisms within ITS region of the rRNA gene.

## Materials and methods

### Nematode samples

Two populations of *Litylenchus crenatae* were used in the present study. The first population was collected in Stone Valley Forest (March 2025), Barree Township, Pennsylvania (GPS coordinates: 40°37’30.3“N, 77°54’06.2”W). The second population was found from an American beech in a forest in New York, USA in October 2019 by Dr. I. Munck (GPS coordinates: 40°59’52.83”N, 73°52’03.14”W 4W). Ten to twenty symptomatic leaves were collected from five trees at each location and sent to the CDFA Nematology Laboratory. Several hundred juvenile stages and adults of *L. crenatae* were extracted from each leaf samples using a standard Baermann funnel method. Extracted nematodes were molecularly identified. DNA of several other nematodes were also used for the specificity assay (Table 1).

**Table 1:** Samples of *Litylenchus crenatae* and other nematodes tested in the present study.

Species	Sample codes	Locations	RPA experiment
*Litylenchus crenatae*	Pen	USA, Pennsylvania	+*
*L. crenatae*	CD4200	USA, New York	+
*Allantonema mirabile*	CD4590	Russia, Moscow region	–
*Anguina agropyri*	CD4588	Russia, Moscow region	–
*A. agrostis*	CD4194	USA, Wyoming	–
*A. graminis*	CD4196	Russia, Moscow region	–
*A. tritici*	CD4189	Iran	–
*Cotylenchus cleo*	CD3762	USA, Washington	–
*Ditylenchus dipsaci*	CD4198	Russia, Kaluga region	–
*Ditylenchoides destructor*	CD4199	Russia, Moscow region	–
*Heteroanguina ferulae*	CD4204	Uzbekistan	–
*Mesoanguina millefolii*	CD4197	Russia, Moscow region	–
*M. picridis*	CD4232	Uzbekistan	–

* + - test line; – no test line.

### Preparation of nematode DNA and crude extract

Nematode DNA, crude extracts and DNA from plant tissues were used for development and validation of the LF-RPA diagnostic assay. A single DNA extraction was performed on pooled nematode specimens using the proteinase K protocol as described by Subbotin et al. [11]. For nematode crude extracts, live nematode specimens were placed into a drop of distilled water on a glass slide and cut by a stainless-steel dental needle under a stereo microscope. Cut nematodes were transferred in a water suspension into a 0.2 mL PCR tube. Two crude extracts, each from 25 nematode specimens in 25 µL of water were used to make a series of two-fold sequential dilutions to test the analytical sensitivity of the assay. DNA from beech leaves with nematodes was extracted using the Qiagen DNeasy Plant Mini Kit (Qiagen, USA) following the manufacturer’s protocol. Total DNA was eluted in a final volume of 30 µL elution buffer and stored at −20ºC. Leaf tissues (0.01 g) were used for each extraction. For the evaluation of the matrix effect, 1, 5, 15, and 30 nematodes were added into tubes with uninfected beech leaves (0.01 g), and then, used for DNA extraction as described above.

### RPA primer and probe design and testing

Two forward and six reverse RPA primers and one probe putatively specific to the beech leaf nematode were proposed based on sequence differences in the ITS rRNA gene between *L. crenatae* and related nematode species (Table 1). Primers were synthesized by Integrated DNA Technologies, Inc. (Redwood City, CA, USA) and then they were tested in different combinations using the TwistAmp® Basic kit (TwistDx, Cambridge, UK). Reactions were prepared according to the manufacturer’s instructions. The lyophilized reaction pellets were suspended in 29.5 µL of rehydration buffer, 2.4 µL each of forward and reverse primers (10 µM) (Table 2), 1 µL of DNA template or nematode extract and 12.2 µL of distilled water. For each sample, 2.5 µL of 280 mM magnesium acetate was added to the lid of the tube. The tubes were inverted 10–15 times, centrifuged and put in a MyBlock Mini Dry Bath (Benchmark Scientific, Sayreville, NJ, USA) at 39ºC. After 4 min, the tubes were again inverted 10–15 times, briefly centrifuged and returned to the incubator block (39ºC) for 20 min. Amplification products were purified with a QIAquick PCR Purification Kit (Qiagen, Germantown, MD, USA). Five microliters of purified product were run in a 1% TAE buffered agarose gel and visualized with a Gel Green stain. Several primers set were selected based on amplification performance.

**Table 2:** Species specific primers and probe for *Litylenchus crenatae* used in the present study.

Code	Sequence (5’ > 3’)
LcF1spec-mod LcR5	AGC AGT TGT ATG GCT TAC AGC CTG TAC CTA AAT TAG CAA AGG CGT GAA
LcR5-biotin	[Biotin] TAC CTA AAT TAG CAA AGG CGT GAA
Probe-Lc1-nfo	[FAM]* AGA ATC AAT GAG TAC CAG CAA GGT GCC GCC [THF] ACA AAA AAC CTC ATT [C3-spacer]

* FAM – fluorophore, THF – tetrahydrofuran, C3 - spacer block.

### LF-RPA assay

The LF-RPA assay was performed using AmplifyRP® Acceler8® Discovery Kit (Agdia, IN, USA). For this assay, reverse primer with biotin labeling and probe were synthesized by Biosearch Technologies (Novato, CA, USA). The reaction mixture for each RPA assay was prepared as recommended by the manufacturer’s instructions: The lyophilized reaction pellet was suspended with a mixture containing 6 µL of the rehydration buffer, 2 µL of distilled water, 0.45 µL each of forward and reverse primers (10 µM), 0.15 µL of the probe (10 µM) and 0.5 µL of magnesium acetate. One microliter of the DNA template or nematode extract was added to a reaction tube. The reaction tubes were put in a MyBlock Mini Dry Bath and incubated at 39ºC in for 20 min. For visual analysis with Milenia® Genline Hybridetect-1 strips (Milenia Biotec GmbH, Giessen, Germany), 120 µL of HybriDetect assay buffer was added to a reaction tube, and then a dipstick was placed in this mixture. Visual results were observed within 2–4 min and then photographed. The amplification product was indicated by the development of a colored test line (lower) and/or a separate control line (upper) to confirm that the system worked properly. Two replicates were performed for sensitivity experiments.

## Results

### RPA detection

**Fig 2:**
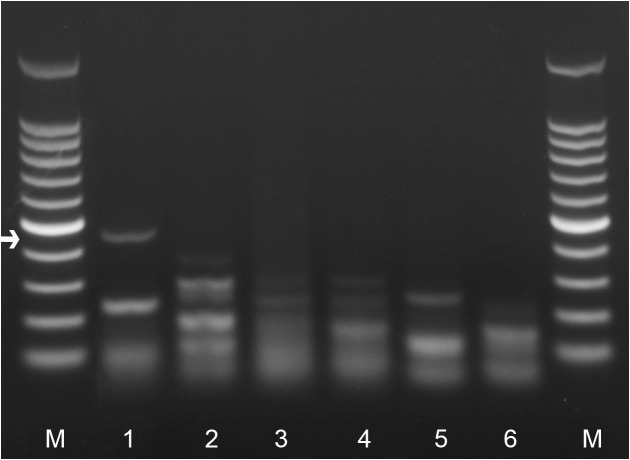
RPA products on agarose gel amplified with LcF1spec-mod and LcR5 using TwistAmp® Basic kit. Lanes: 1*: L*. *crenatae* (Pen); 2: *Anguina agrostis* (CD4194); 3: *A. graminis* (CD4196); 4: *Mesoanguina millefolii* (CD4197); 5: *Ditylenchus dipsaci* (CD4198); 6: negative control (no DNA); M: 100 bp DNA marker (Promega, Madison, WI. USA). Species-specific amplicon are indicated by an arrow.

**Fig 3:**
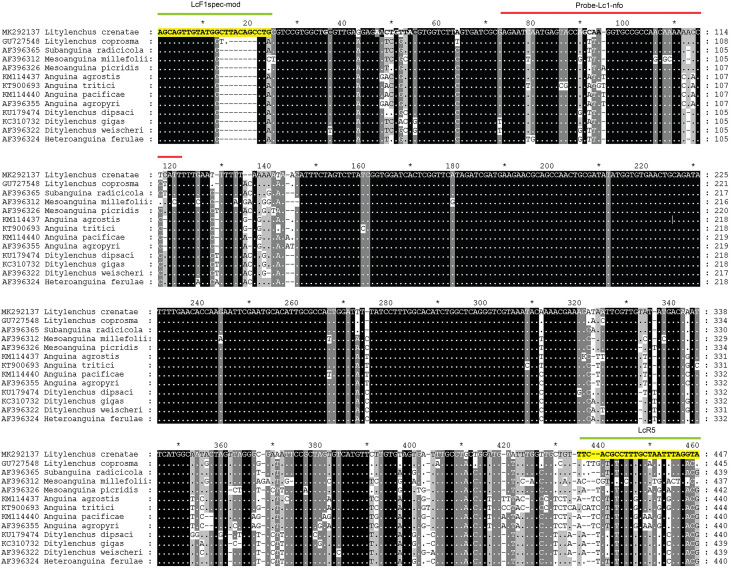
The fragment of the ITS rRNA gene sequence alignment of *Litylenchus crenatae* and some related anguinid nematodes with positions of RPA primers (yellow) and probe used in the present assay.

Several primer combinations were tested for the best performance under the same RPA conditions. The species-specific forward LcF1spec-mod and reverse LcR5 primers were selected as optimal, with a clearly visible specific band using the TwistAmp® Basic. This primer set reliably and specifically amplified the target gene fragment approximately 447 bp in length on a gel (Fig 2). No-specific amplification resulting fragments of different lengths were observed in non-target samples and control. The specific primers and probe sequences used for the assay are listed in Table 2 and are indicated in the ITS rRNA gene alignment in Fig 3.

### Specificity of LF- RPA assay

**Fig 4:**
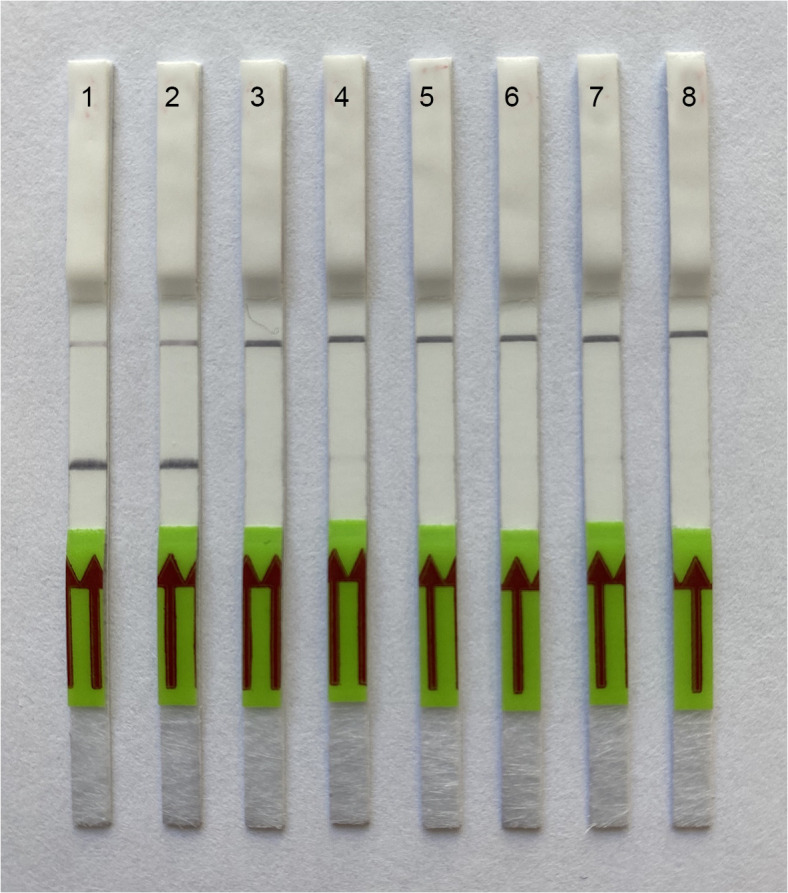
Lateral flow recombinase polymerase amplification (LF-RPA) assay with examples of lateral flow strips. Specificity assay with DNA samples of different nematodes. Strips: 1: *Litylenchus crenatae* (Pen); 2: *L. crenatae* (CD4200); 3: *Anguina graminis* (CD4196); 4: *Mesoanguina millefolii* (CD4197); 5: *Ditylenchoides destructor* (CD4199); 6: *Ditylenchus dipsaci* (CD4198); 7: *Anguina agropyri* (CD4588); 8: negative control (no DNA).

Nematode DNA and crude extracts from two populations of *L. crenatae,* ten species of plant parasitic nematodes from the family Anguinidae and entomoparasitic nematode, *Allantonema mirabile* were tested in this assay (Table 1). The RPA results showed high specificity to *L. crenatae* only, and no positive bands were observed in samples with any other nematodes. Positive test lines on the LF strips were observed for *L. crenatae* samples within 2–4 min, whereas the samples with other nematode species showed only a control line (Fig 4). In some experiments, very weak test bands on LF strips with negative controls and non-target DNA samples might be observed in 15 and more minutes after adding HybriDetect assay buffer.

### Sensitivity of LF- RPA assay

**Fig 5:**
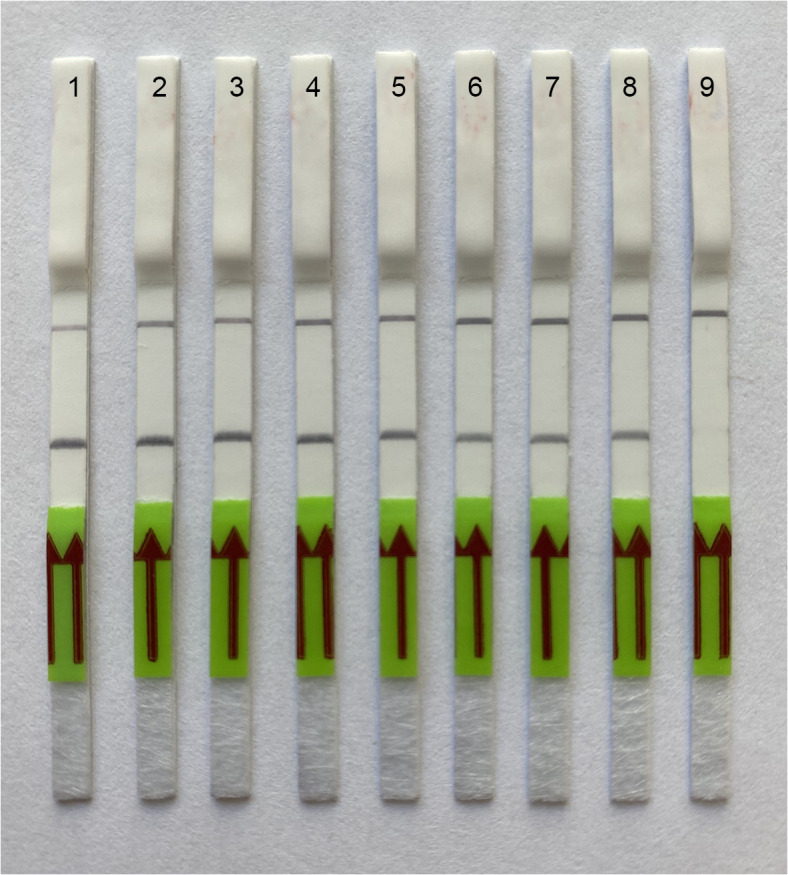
Lateral flow recombinase polymerase amplification (LF-RPA) assay with examples of lateral flow strips. Sensitivity assay with crude nematode extract of *Litylenchus crenatae* (CD4200). Strips: 1: 1 nematode per tube reaction; 2: 0,5 nematode per tube; 3: 0.25 nematode per tube; 4: 0.125 nematode per tube; 5: 0.06 nematode per tube; 6: 0.015 nematode per tube; 7: 0.007 nematode per tube; 8: 0.002 nematode per tube; 9: negative control (no DNA).

**Fig 6:**
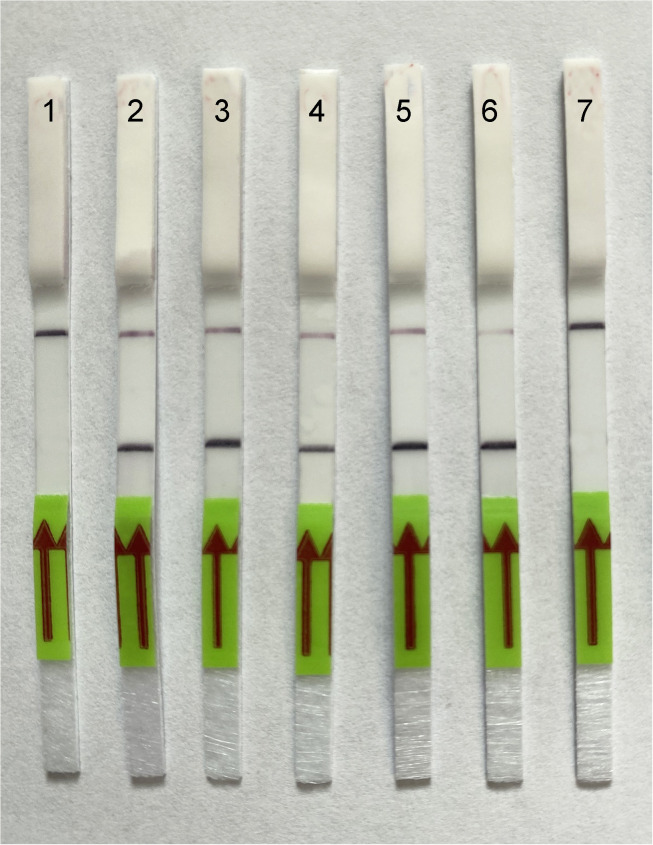
Lateral flow recombinase polymerase amplification (LF-RPA) assay with examples of lateral flow strips. Sensitivity assay with DNA extracted from samples containing beach leaves and different numbers of *Litylenchus crenatae* (CD4200) specimens. Strips: 1: 0.01 g beech leaf without nematodes (negative control); 2: 1 µL from 30 µL DNA extract from 0.01 g beech leaf + 1 nematode; 3: 1 µL from 30 µL DNA extract from 0.01 g beech leaf + 5 nematodes; 4: 1 µL from 30 µL DNA extract from 0.01 g beech leaf + 15 nematodes; 5: 1 µL from 30 µL DNA extract from 0.01 g beech leaf + 30 nematodes; 6: nematode crude extract (positive); 7: negative control (no DNA).

The analytical sensitivity assay estimated the specimen number detection limit for a crude nematode extract and for DNA extracted from beech leaves with nematodes. Two-fold serial dilutions of crude nematode extract were prepared with a range between 0.5 and 0.002 nematode per tube. Strong band on a strip was observed in variants with 0.002 nematode per tube (Fig 5). DNA extracted from 0.01 g of healthy beech leaves with 1, 5, 15 and 30 nematodes was used in another study. RPA assays reliably detected this nematode species with plant DNA extracted from 0.01 g of healthy beech leaves containing 1 or more nematode specimens per tube with DNA extraction or 0.03 nematode per reaction tube (Fig 6).

## Discussions and conclusions

In this study, we developed an affordable, simple, rapid, and highly sensitive lateral flow recombinase polymerase amplification (LF-RPA) assay for the detection of *Litylenchus crenatae* from symptomatic beech leaf disease samples. The LF-RPA assay can be performed entirely under field conditions without the need for specialized laboratory equipment, making it suitable for on-site diagnostics and large-scale disease surveillance. The integration of this primer-probe set into the LF-RPA platform provides a reliable, field-deployable molecular tool for early and accurate identification of *L. crenatae*, supporting rapid disease management decisions and improving understanding of BLD epidemiology.

Species-specific primers and probes were designed based on the ITS1–5.8S rRNA–ITS2 region of the *L. crenatae* rRNA gene cluster. The forward primer LcF1spec-mod used in this study was designed to target a unique insertion within the ITS1 region that is specific to *L. crenatae*. This distinctive sequence feature was previously utilized by Burke et al*.* [7] to design the 234R reverse primer, which enhances the specificity of detection for this nematode species. Together, these primers amplify distinct DNA fragments that are unique to *L. crenatae*, minimizing the risk of cross-reactivity with closely related species.

Our study estimated that the sensitivity of RPA assays using lateral flow dipsticks, the detection threshold was approximately 0.002 nematode per reaction tube and 0.03 nematode per reaction tube from DNA extract obtained from beech leaves. RPA-based diagnostic for the beech leaf nematode offers several significant advantages over currently used conventional PCR methods. One key benefit is that crude extracts from nematodes can be directly used in RPA assays, eliminating the need for DNA extraction and specialized samples required for PCR. The complete detection process using the LF-RPA assay can be accomplished in approximately 30 minutes, in contrast to the 1.5 to 3 hours typically needed for PCR analysis. This includes 4 minutes for crude nematode extract preparation, 20 minutes for the RPA reaction, 1 minute for mixing and centrifugation, and 3–5 minutes for visual detection using lateral flow strips. RPA also demonstrates superior sensitivity, being 10- to 100-fold more sensitive than PCR. Despite these advantages, the broader application of RPA in nematode diagnostics may be limited by certain challenges, particularly the relatively high cost of reagents and materials, as well as the very limited number of companies that manufacture these kits.
